# Integrated Information and State Differentiation

**DOI:** 10.3389/fpsyg.2016.00926

**Published:** 2016-06-28

**Authors:** William Marshall, Jaime Gomez-Ramirez, Giulio Tononi

**Affiliations:** Department of Psychiatry, Center for Sleep and Consciousness, University of WisconsinMadison, WI, USA

**Keywords:** integrated information theory, state differentiation, cause-effect power, animats, consciousness

## Abstract

Integrated information (Φ) is a measure of the cause-effect power of a physical system. This paper investigates the relationship between Φ as defined in Integrated Information Theory and state differentiation (D), the number of, and difference between potential system states. Here we provide theoretical justification of the relationship between Φ and D, then validate the results using a simulation study. First, we show that a physical system in a state with high Φ necessarily has many elements and specifies many causal relationships. Furthermore, if the average value of integrated information across all states is high, the system must also have high differentiation. Next, we explore the use of D as a proxy for Φ using artificial networks, evolved to have integrated structures. The results show a positive linear relationship between Φ and D for multiple network sizes and connectivity patterns. Finally we investigate the differentiation evoked by sensory inputs and show that, under certain conditions, it is possible to estimate integrated information without a direct perturbation of its internal elements. In concluding, we discuss the need for further validation on larger networks and explore the potential applications of this work to the empirical study of consciousness, especially concerning the practical estimation of Φ from neuroimaging data.

## Introduction

Integrated information (Φ) is a quantity that describes the intrinsic causal properties of a physical system in a state (Oizumi et al., 2014). Integration emphasizes that a unitary system must have causal power beyond its constituent parts. Information emphasizes that the causal power of a system in a state must be specific, implying that it should be different in different states, a property also referred to as differentiation (Tononi and Edelman, 1998; Tononi, 2004). Despite this intuitive connection, a formal examination of the relationship between integrated information and differentiation has not yet been performed. Here, we explore the notion of state differentiation (D) explicitly, propose a way to measure it, and assess its relationship to the integrated information of a physical system in a state.

Integrated information has been used as a measure of complexity in dynamical systems, such as cellular automata (Albantakis and Tononi, 2015) and artificially evolved agents (animats) (Edlund et al., 2011; Albantakis et al., 2014). Moreover, integrated information theory (IIT) relates, Φ to the level of consciousness in a physical system, since integration (unity) and specificity (differentiation) are two fundamental properties of experience (Tononi, 2004; Oizumi et al., 2014). The connection between consciousness and integrated information proposed by IIT has motivated neuroimaging studies which employ simple perturbational approaches aimed at estimating the brain's capacity to integrate information (Massimini et al., 2005; Casali et al., 2013).

While IIT makes many interesting predictions relating Φ and the neural substrate of consciousness (Tononi, 2012; Oizumi et al., 2014; Tononi and Koch, 2015), both the theory and some of its counterintuitive predictions are controversial (Aaronson, 2014; Barrett, 2014; Dehaene et al., 2014). To validate these predictions, one should ideally evaluate Φ on real neuronal systems; however, this is not currently possible except using proxy measures, such as differentiation, which motivates the present contribution. There are two practical issues which must be overcome before Φ can be calculated for a broader class of systems. First, the calculation requires a proper causal model of the system, as could be obtained by perturbing the elements of the system into all possible states. Second, it requires evaluating all possible bi-partitions of the system, a task that is computationally infeasible for moderate to large sized systems. Theoretical advances and new experimental procedures may eventually allow us to exhaustively perturb and partition a system, but until then it is not possible to apply integrated information for even modestly sized physical systems.

One alternative that has been pursued is to develop empirical measures that quantify the balance between differentiation and integration that characterizes physical systems with high Φ. Neural complexity was the first attempt in this direction, using mutual information between parts of a system (Tononi et al., 1996). A more recent measure, causal density (Seth, 2005), utilizes a similar approach but applies the statistical notion of Granger causality to quantify the integration of directed causal interactions within a system. Adaptations of Φ for time series data have also been suggested, such as empirical Phi (Φ_*E*_) (Barrett and Seth, 2011) and Phi star (Φ^*^) (Oizumi et al., 2015).

Here we explore the possibility of estimating Φ by using measures of state differentiation. We will demonstrate theoretically, that state differentiation can be successfully used to assess the intrinsic cause-effect power of the brain (and similar systems) in practice, because there is a strong relationship between Φ and D, if one can assume that the system of interest is integrated and connected effectively to an external environment. Measures of differentiation are easier and faster to compute than other approximations of integrated information, and can be readily employed in neuroimaging studies (Gosseries et al., 2011; Sarà et al., 2011; Barttfeld et al., 2015; Boly et al., 2015; Hudetz et al., 2015; Montijn et al., 2016).

The goal of this work is to elucidate the relationship between Φ and D, providing the theoretical foundation for future empirical studies to use state differentiation as a proxy for the intrinsic cause-effect power of a system. The rest of the paper is structured as follows. In Section 2 we formally define integrated information and differentiation, and then derive their theoretical relationship. Section 3 presents the results of simulation studies, further explicating the relationship between Φ and D. Finally, in Section 4 we discuss the current work in the context of neuroimaging studies of consciousness and highlight points to be considered for future studies.

## Methods

Complete expositions of IIT are provided in Oizumi et al. (2014) and Tononi (2015). Here, focus is given to aspects of the theory necessary to understand the current results. In the mathematical definitions, we use the convention that a bold faced character represents a vector. Integrated information is evaluated on a *physical system*, which is a collection of *elements* in a state, at a specific spatio-temporal grain. Elements are units having at least two possible states, inputs which affect its state and outputs which are affected by its state. Additionally, it is possible to manipulate, observe and partition physical elements. By randomly setting the system into all possible states according to a maximum entropy distribution, and observing the subsequent state transitions, while holding elements outside the physical system fixed (Oizumi et al., 2014), we are able to describe the causal properties of the system (Pearl, 2009). In the current work, we represent a physical system by a discrete random vector **S** = *S*_1_, *S*_2_, …, *S*_*n*_, where each *S*_*i*_ is an element of the system, and the causal properties by a transition probability function *p* : Ω_*S*_ × Ω_*S*_ → (0, 1) which describes its state-to-state transitions. In what follows, we focus on a physical system **S** consisting of *n* binary elements, and state space $\Omega_{S}={\left\{0,\;1\right\}}^{n}$, with $|\Omega_{S}|=2^{n}$ states, although results are expected to extend to non-binary elements. However, the assumption is that each of these mathematical representations corresponds to a real physical system, and that the transition probability function has been determined from a systematic perturbation of system elements.

There are five postulates that IIT employs to characterize the cause-effect power of physical systems: existence, composition, information, integration and exclusion. Here we elaborate on the application of these postulates to characterize a physical system in a state and its intrinsic causal properties.

### Mechanisms

#### Existence and composition

The notion of causality is fundamental to explain the nature of a physical system. A set of elements that forms a causal relationship with other elements of the physical system is called a mechanism. A mechanism must have both causes and effects within the physical system. In IIT the basis for analyzing causal relationships is the *cause repertoire* and *effect repertoire*.

A repertoire is a probability distribution which describes the possible past or future states of a set of elements in the physical system, as constrained by the current state of another (potentially different) set of elements. The set of elements whose potential states are described by a cause or effect repertoire is called its purview (**Z**), and the set of elements that constrain the purview is called the candidate *mechanism* (**M** = **m**_t_). The purview and candidate mechanism are compositions of elements within the system, i.e., they are any subsets of the physical system. For a system of *n* binary elements, this means that there are 2^n^-1 candidate mechanisms for the system. A cause repertoire describes the possible states of a past purview **Z**_*t*−1_,

$$p_{\text{cause}}(z|m_{t}),\;z\in \Omega_{Z_{t-1}},$$

while an effect repertoire describes the possible future states of a future purview **Z**_*t*+1_,

$$p_{\text{effect}}(z|m_{t}),\;z\in \Omega_{Z_{t+1}}.$$

Note that these probability distribution are not obtained by simply observing the system. In order to measure the causes and effects a perturbation analysis must be performed, similar to applying the *do()* operator defined by Pearl (2009). For full details of the repertoire calculation, refer to Appendix S1.

#### Information

The *unconstrained repertoires* are probability distributions over the potential past and future states of a physical system with no constraints from the current state of the elements. This corresponds to applying a maximum entropy perturbation to the output of the purview to obtain the cause repertoire, and to the inputs of the purview to obtain the effect repertoire. The result is a maximum entropy distribution on the cause side, while the effect side describes the one-step transition probabilities of the physical system.

The cause-effect information of a mechanism resides precisely in its capacity to specify the past and future states of the system. To measure the cause-effect information of a mechanism in a state, its causes and effects are quantified using the *earth movers distance* (EMD) metric between two probability distributions (see Appendix S1). The cause (effect) information of a mechanism in a state is the distance between its constrained repertoire and the corresponding unconstrained repertoire,

$$ci(m_{t})=\text{emd}(p_{\text{cause}}(z|m_{t}),p_{\text{cause}}(z|\emptyset )),$$

$$ei(m_{t})=\text{emd}(p_{\text{effect}}(z|m_{t}),p_{\text{effect}}(z|\emptyset )).$$

In summary, the cause and effect information quantify how the current state of a mechanism constrains the possible past and future states of the system. A mechanism must constrain both the past and future states of the system, i.e., have both causes and effects. The cause-effect information of a mechanism is the minimum of its cause and effect information, *min*(*ci, ei*).

The larger the state space of the system, the more potential there is for a mechanism to constrain the possible past and future states. This idea is formalized in the following theorem,

**Theorem 2.1.** *For a physical system in a state* **S** = **s**_*t*_ ∈ Ω_*S*_ *with n binary elements, the cause and effect information of a mechanism in a state* ***M*** = ***m***_*t*_ *are bounded*,

$$ci(m_{t})\;\leq \;\frac{n}{2}=\frac{\log_{2}|\Omega_{S}|}{2},\text{ and }ei(m_{t})\;<\;n=\log_{2}|\Omega_{S}|.$$

***Proof:** See Appendix S2*.

Theorem 2.1 provides the first link between IIT and the state space of a system. The maximum possible cause-effect power of a mechanism is determined by the size of the state space of the physical system. The potential cause-effect information increases logarithmically with the size of the state space of the system. The different results for cause and effect information are related to differences in the unconstrained repertoires; the unconstrained effect repertoire has the potential to be asymmetric, which allows some states to have larger values of effect information (while others must have less).

#### Integration

A mechanism's cause-effect information must also be irreducible. This means that any decrease in the mechanism's connections must result in a loss of cause-effect information. This precludes the inclusion of unnecessary elements that do not contribute to the cause or effect, and prevents the combination of unrelated mechanisms to create a larger mechanism that is nothing more than the sum of its parts.

To assess irreducibility, we consider the information specified by a mechanism above and beyond that of a partition (or *cut*). This irreducible information is quantified by the EMD between the repertoires for the unpartitioned (whole) and partitioned (cut) mechanism. Details on how to apply the cut, and find the partitioned repertoire are given in Appendix S1. The cut that makes the least difference to the mechanism is called its *minimum information partition* (MIP),

$$\text{MIP}=\underset{\text{cut}}{\arg\min}\left\{\text{emd}\left(p_{\text{cause}}(z|m_{t}),p_{\text{cause}}^{\text{cut}}(z|m_{t})\right)\right\}.$$

The irreducible cause-effect power of a mechanism is measure by its *integrated information* (φ), the information generated by the whole above and beyond its MIP. The integrated cause (effect) information of a mechanism is,

$$\varphi (m_{t},Z_{t-1})=\text{emd}(p_{\text{cause}}(z|m_{t}),p_{\text{cause}}^{\text{MIP}}(z|m_{t}))$$

$$\varphi (m_{t},Z_{t+1})=\text{emd}(p_{\text{effect}}(z|m_{t}),p_{\text{effect}}^{\text{MIP}}(z|m_{t}))$$

#### Exclusion

Finally, cause-effect power should not be counted multiple times. Thus, given that each mechanism has only one cause and one effect, they are defined as the ones which maximize cause-effect power. To find the cause-effect power of a mechanism, the integrated information is evaluated across all possible purviews, to find the ones that are maximally irreducible.

$$\varphi_{\text{cause}}^{\max}(m_{t})=\underset{Z_{t-1}}{\max}\left\{\text{emd}(p_{\text{cause}}(z|m_{t}),p_{\text{cause}}^{\text{MIP}}(z|m_{t}))\right\}.$$

$$\varphi_{\text{effect}}^{\max}(m_{t})=\underset{Z_{t+1}}{\max}\left\{\text{emd}(p_{\text{effect}}(z|m_{t}),p_{\text{effect}}^{\text{MIP}}(z|m_{t}))\right\}.$$

A mechanism is irreducible only if both its cause information and effect information are irreducible. The integrated information of **m**_*t*_ is the minimum of its irreducible cause and effect information,

$$\varphi^{\max}(m_{t})=\min\left\{\varphi_{\text{cause}}^{\max}(m_{t}),\varphi_{\text{effect}}^{\max}(m_{t})\right\}.$$

If a candidate mechanisms cause-effect information is completely reducible, φ^max^ = 0, then it is not a mechanism.

**Corollary 2.2.** *For a physical system in a state* **S** = **s**_*t*_ ∈ Ω_*S*_ *with n binary elements, and mechanism **M** = **m**_t_, the integrated information of **m**_t_ is bounded by*

$$\varphi^{\max}(m_{t})\leq \frac{n}{2}.$$

***Proof:** See Appendix S2*.

Corollary 2.2. strengthens the link between IIT and the state space described in Theorem 2.1. The integrated information of a mechanism is bounded by the cause-effect information, and thus the size of the state space of a physical system. This means that the MIP can never increase the information of a mechanism. The greatest possible effect is that a partition of the mechanism eliminates all information about the past and future states of the purview, and in this case the integrated information is equal to the cause-effect information of the mechanism.

### Physical systems

So far, we have used the postulates of IIT to define the mechanisms within a system **S**. We are now in a position to consider the system as a whole.

#### Information

For a physical system in a state, the cause-effect structure of the system is the set of mechanisms with irreducible cause-effect power (φ^max^ > 0) and corresponding cause-effect repertoires,

$$C(s_{t})=\{(m_{t},p_{\text{cause}},p_{\text{effect}})|\varphi^{\max}(m_{t})>0\}.$$

To have cause-effect power, a system must have at least one mechanism in its cause-effect structure. The more mechanisms a system has in its cause-effect structure, both in number and magnitude (φ), the more cause-effect power the system can have. Since the empty set necessarily has φ^max^ = 0, the maximum number of mechanisms in the cause-effect structure of **S** is one less than the size of its power set,

$$|C(s)|\leq \sum_{i=1}^{n}\left(\begin{array}{c} n\\ i \end{array}\right)=2^{n}-1,\;s\in \Omega_{S}.$$

The number of mechanisms, and thus the total cause-effect power of a cause-effect structure is constrained by the number of elements which constitute the physical system. The maximum number of mechanisms increases exponentially with the size of the physical system.

#### Integration

Analogous to the irreducibility of mechanisms, physical systems must be irreducible to their constituting parts. To assess irreducibility at the systems level, we consider the effect of a directed partition (or *system cut* on the cause-effect structure. The effect of the system cut is measured by using the EMD to compute the distance between cause-effect structures (see Appendix S1 for details).

A directed partition of a physical system is a partition of the system elements into two subsets, with the connections from the first subset to the second cut (injected with noise). The MIP of a physical system is the directed partition which makes the least difference, i.e., it minimizes the distance between cut and uncut cause-effect structures,

$$\text{MIP}=\underset{cut}{\arg\min}\left\{\text{emd}(C(s_{t}),C(s_{t}^{\text{cut}}))\right\}.$$

The integrated information (Φ) of a physical system is measured by evaluating the distance between its cause-effect structure and the cause-effect structure of its MIP. Using the MIP to evaluate integrated information amounts to cutting the “weakest link” of the system, so that including unnecessary elements in the physical system will reduce the integrated information,

$$\Phi (s_{t})=\text{emd}(C(s_{t}),C(s_{t}^{\text{MIP}}))$$

#### Exclusion

Once again, only maximally irreducible systems are considered. This is because a mechanism can only have one cause and one effect, and thus can only contribute to one cause-effect structure without counting causes and effects double, and that is the cause-effect structure which is maximally irreducible,

$$\Phi =\Phi^{\text{max}}(s_{t})=\{\begin{array}{l} \Phi (s_{t})\text{ if }\Phi (s_{t}^{*})<\Phi (s_{t}),\;\forall (S^{*}\cap S)\neq \emptyset \\ 0\text{ otherwise} \end{array}$$

The set of elements with the maximally irreducible cause-effect structure is called the *complex* of a physical system. Next we extend the previous result to show how system size also bounds the integrated information of a system.

**Theorem 2.3.** *For a physical system in a state* **S** = **s**_*t*_ ∈ Ω_*S*_ *with n binary elements, the integrated information of the cause-effect structure of its complex is bound by*

$$\Phi^{\text{max}}(s_{t})\leq \frac{(2^{n}-1)3n^{2}}{4}.$$

**Proof:** See Appendix S2.

The maximum possible information corresponds to when all potential mechanisms are included, and specify the maximum amount of cause and effect information. The maximum possible integration is when all information specified by the mechanisms is lost after the MIP.

Theorem 2.3 establishes a key result for this work: systems with high Φ must have a large number of elements and mechanisms, and thus a large state space. For a physical system with a fixed number of elements, as the value of Φ increases, so must the number of mechanisms. It now remains to connect this result to the notion of differentiation.

### Differentiation

The differentiation of a physical system is the diversity of its potential states. Here we consider two measures of differentiation that can be mathematically related to integrated information. Both measures of differentiation are calculated from the transition probably function, and thus require a causal analysis of the elements constituting the physical system (internal perturbation). Evoked differentiation using external perturbation through stimuli manipulation is explored in the Results section.

The first measure of differentiation (D_1_) is the number of potential states of the system, i.e., the number of states which could occur following every possible state the system can be perturbed into,

$$D_{1}=\left|\left\{s\;|\;\sum_{s^{*}\in \Omega_{S}}p(s^{*},s)>0\right\}\right|.$$

In other words, D_1_ is the number of states that the system can potentially transition into from all other states within the state space. This is different from the size of the state space of a physical system, which is always $|\Omega_{S}|=2^{n}$. The number of potential states may be less than the size of the state space in the case of convergence; it depends on what the system actually does.

**Figure 1:**
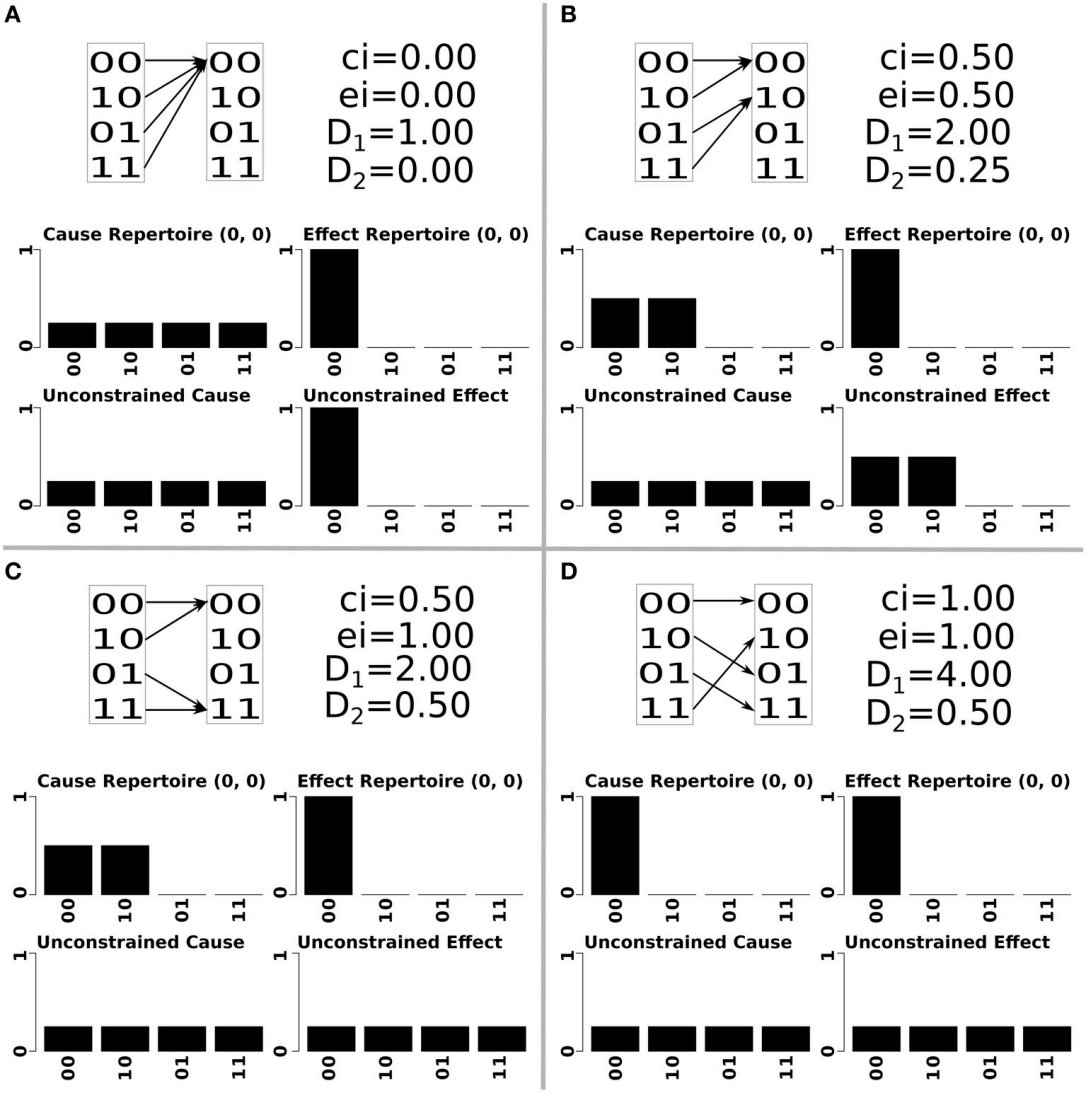
**An illustration of two element systems with increasing values of differentiation (note that the system elements displayed here are only a subset of a larger set of elements)**. Each quadrant of the figure shows the state transitions and differentiation values of the corresponding system, as well as the constrained and unconstrained cause-effect repertoire and cause-effect information of the mechanism composed of both elements, in the state **m** = (0, 0). System **(A)** has no differentiation, system **(B)** has low values of both D_1_ and D_2_, system **(C)** has low D_1_ and high D_2_, system **(D)** has high values of D_1_ and D_2_. As D_1_ increases (systems *A*→*B*→*D*), so does the cause information of the mechanism. This change happens by making the mechanism's cause repertoire more selective without changing the unconstrained cause repertoire (reducing convergence). As D_2_ increases (systems *A*→*B*→*C*), so does the effect information of the mechanism. This happens by increasing the entropy of the unconstrained effect repertoire without changing the effect repertoire (reducing divergence).

There are two ways in which D_1_ affects Φ, both relating to cause information. First, states without a cause have no cause information, no mechanisms and thus Φ = 0. For a deterministic system, every state that is impossible means that there is a different state with an additional cause. This decreases the selectivity, i.e., increases degeneracy, of that state (a completely degenerate state could have come from any previous state, while a non-degenerate state has a unique cause) which corresponds to less cause information, lower φ values for mechanisms and lower Φ values of the system (Hoel et al., 2013; Albantakis and Tononi, 2015). This can be seen in Figure 1, where increasing the number of potential states that can be reached increases the amount of cause information. The cause information increases from 0 to 0.5 to 1, as the number of potential states increases from 1 to 2 to 4 Figures 1A,B,D. As the unconstrained cause repertoire remains the same for all panels, it is the increased selectivity of the cause repertoire from four possible causes to just one, that results in higher values of cause information.

The D_1_ measure captures the number of potential states, but there is another aspect to differentiation, the differences between those states. The second measure of differentiation (D_2_) is the cumulative variance of all the elements of the system,

$$D_{2}=\sum_{i=1}^{n}u_{i}(1-u_{i}),$$

where *u*_*i*_ is the unconstrained probability element *S*_*i*_ will be in the ON (1) state given all possible perturbations of its inputs,

$$u_{i}=p_{\text{effect}}(s_{i,t}=1|\emptyset ).$$

This measure captures how different the potential states of the system are from each other. The most different two binary states can be, is for them to have the opposite state for each element, e.g., state (0, 0, 0) to state (1, 1, 1) in a three element system. The average distance between states is then maximized when each element is equally likely to be ON (1) or OFF (0) in the next state. For each element, the variance of its potential state is *u*_*i*_(1 − *u*_*i*_), and this quantity reaches its maximum value when *u*_*i*_ = 0.5 (maximum entropy).

The D_2_ measure relates to integrated information via the effect information of mechanisms. The unconstrained effect repertoire is calculated by independently injecting maximum entropy into the inputs of each element (see Appendix S1), so its entropy is equal to the sum of the entropy of the corresponding elements. Since effect information of a mechanism is the distance between the constrained and unconstrained effect repertoire, an unconstrained effect repertoire with greater entropy has more potential average effect information. Larger values of D_2_ correspond to greater entropy and allow for more potential average effect information and thus more potential average integrated information.

In Figure 1, as D_2_ increases from 0 to 0.25 to 0.5, the effect information increases from 0 to 0.25 to 1 Figures 1A–C. In this situation the effect repertoire has the same selectivity in all panels, and it is the increasing entropy of the unconstrained effect repertoire which permits higher values of effect information.

The above intuition about the relationship between D_1_, D_2_ and cause-effect information is formalized in the following theorem,

**Theorem 2.4.** *For a physical system **S** with n binary elements, state space Ω_S_ and candidate mechanism* **M** ⊆ **S** *the average cause and effect information of **M** are bounded*,

$$\mu [ci(m)]\;\leq \;\frac{nD_{1}}{2^{n+1}}\;\leq \;\frac{n}{2},\text{ and }\mu [ei(m)]\;\leq \;2D_{2}\;\leq \;\frac{n}{2}.$$

***Proof:** See Appendix S2*.

Theorem 2.4 directly relates the notion of differentiation to the average cause and effect information, quantities that are fundamental in IIT. The result allows for the possibility that a system with low values of D_1_ or D_2_ could have large values of cause-effect information for a single state, but then other states must have low cause-effect information. Only a system with large values of D_1_ and D_2_ can have high values of cause-effect information in most states. The average cause information is bounded by the number of accessible states in the system, and this bound can be achieved only if the system is non-degenerate, so that each potential state has a unique cause. The effect information is bounded by the cumulative entropy of individual element states, and this bound is maximized when the entropy of each individual element is maximized. A mechanism can only reach the upper bound of effect information if the system is deterministic, so that the constrained elements have minimal (zero) entropy and the distance between constrained and unconstrained repertoires is as large as possible. This reinforces the result of Albantakis and Tononi (2015) and Hoel et al. (2013), that determinism and non-degeneracy are properties of systems with larger values of integrated information.

The following theorem provides the principal theoretical result of this work, providing a direct link between differentiation and integrated information,

**Theorem 2.5.** For a physical system **S** with *n* binary elements and corresponding state space $\Omega_{S}={\left\{0,\;1\right\}}^{n}$, the average integrated information is bounded by

$$\mu [\Phi ]\leq (2^{n}-1)\frac{n}{2}\left(\frac{nD_{1}}{2^{n+1}}+2D_{2}\right)\leq \frac{(2^{n}-1)n^{2}}{2}$$

***Proof:** See Appendix S2*.

Theorem 2.5 further constrains the average integrated information than the bound implied by Theorem 2.3. Any reduction in differentiation, either in the number of states (D_1_) or the distance between states (D_2_) results in a reduction of the potential integrated information of the system. To have the maximum potential integrated information, a physical system must be able to enter every state in its state space and each element of the system should be equally likely to be ON (0) or OFF (1) in the future state. The amount of integrated information a system actually has depends on how integrated it is. For example, a pure noise system can visit all possible states, and each element is equally likely, but it will have Φ = 0 because it is not integrated. It should be noted that the bound of Theorem 2.5 assumes that a system, and each of its mechanisms can all be “maximally integrated,” in the sense that all information is lost when the system or mechanism is partitioned. However, this is not possible in practice, so no system will actually reach this upper bound. Future work that further explores integration should be able to provide a tighter bound on μ[Φ].

In summary, if a system has a large value of integrated information then it must be integrated, have many elements, a large state space and many mechanisms. Furthermore, to have a large average value of integrated information, it is not sufficient that there is a large state space, the system must also have the capacity to visit many states (D_1_), and the states it visits should be as distant as possible from each other (D_2_), i.e., it must have high differentiation.

### Typical states

The above results suggest that tractable differentiation quantities may be used as a proxy for the average Φ of a physical system. However, since in IIT integrated information is a state dependent quantity, the following theorem introduces a relationship between the average integrated information of a system and the integrated information of a *typical* state.

**Theorem 2.6.** *Consider the integrated information for a random state of a physical system. If* Φ ∝ ∑φ *and* σ[Φ] = o(μ[Φ]), *then for any* ϵ > 0 *and* δ > 0 *there exists* μ_0_ *such that for all system with* μ[Φ] > μ_0_,

P(|Φ−μ[Φ]|≥δμ[Φ])≤ϵ.

***Proof:** See Appendix S2*.

As the value of μ[Φ] gets very large (and hence the number of elements must increase, see Theorem 2.5), the probability of observing a value of Φ that is relatively different from the average is essentially zero. This means that for large integrated systems, when state specific information is not available we are still able to make useful inferences, such as approximating the integrated information of a specific state from the average Φ of the physical system. The feasibility of the theorem assumptions are discussed in Appendix S2. If satisfied, this allows us to use the average integrated information, and thus the differentiation, as a proxy for the state-dependent integrated information of a physical system.

## Results

The previous section established that greater values of integrated information imply greater levels of differentiation. A natural follow-up question is whether the reverse is true, do high levels of differentiation imply high integrated information? or are there any other relationships between integrated information and differentiation? These questions are further explored in two different settings, using artificially evolved networks called *animats*. The first is a controlled environment, where a full internal perturbational analysis is applied to the elements of the system to calculate Φ and differentiation values exactly. Next is a setting where external perturbation techniques (stimulus manipulation) are used to evoke differentiation.

Animats are artificial entities consisting of several elements, connections between them, and a logic governing the interaction of the elements. There are three types of elements: sensors receive inputs only from the outside world and may send output to other elements, motors may receive inputs from other elements but only send output to the external world, and internal elements have no direct connections with the external world but may receive inputs and send outputs to other elements. The network structure of each animat was evolved by mutation over many generations using a genetic algorithm (Edlund et al., 2011). The population of animats was ranked according to a fitness function; animats with high fitness were more likely to contribute to the next generation than animats with low fitness.

**Figure 2:**
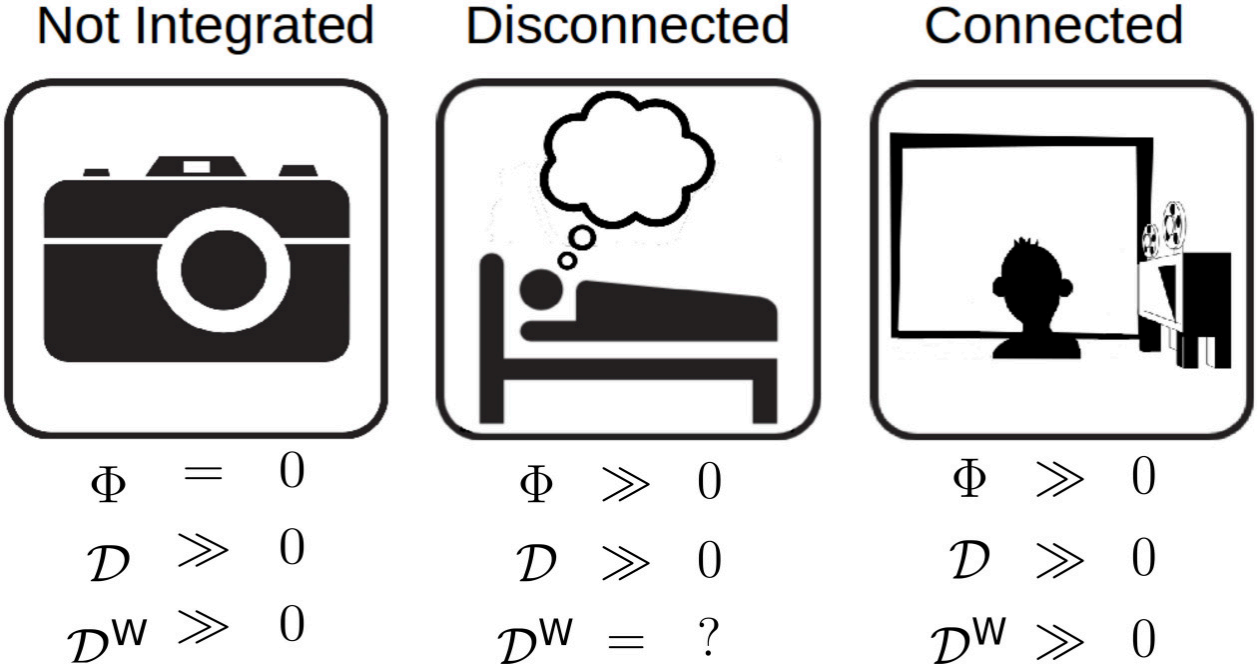
**The relationship between differentiation and Φ for three different classes of system**. Left: A high differentiation system that is not integrated, such as a camera. Although the camera has the potential to enter many states, it has Φ = 0 because it is not integrated. Middle: An integrated system that is disconnected from its environment, such as a dreaming human. Its differentiation is related to Φ; however, since it is disconnected from the environment, external perturbations cannot be used to evoke differentiation. Right: An integrated system that is connected to its environment. Its *D* is also related to Φ, but in addition, it can be influenced to explore its internal structure using rich stimulus sets.

In general, high differentiation does not necessarily imply high integrated information: a system which is not integrated can have high differentiation, but be completely reducible and thus have zero Φ. Thus, a relationship between differentiation and integrated information is only possible if the system can be shown or assumed to be integrated (see Figure 2). To investigate the relationship between D_1_, D_2_ and Φ, a population of animats was evolve to a fitness function that was the integrated information of the most common state of the system during evolution. Two different animat configurations were evolved for this experiment: 2 sensors, 4 hidden units, 2 motors and 3 sensors, 3 hidden units, 2 motors. In total, 56 animats were evolved to have integrated structures, 28 for each element configuration.

To study the differentiation evoked by a stimulus set (D^*W*^) evoked by a stimulus set W, we require a means to present the stimulus to the network (see Figure 2). Of the original 56 animats, there were 10 which received no input from the environment, and they were excluded. The remaining 46 integrated animats were connected to the environment using their sensors, and they were used to explore the relationship between integrated information and stimulus set differentiation.

### Relationship between Φ and D_1_, D_2_

An perturbational approach is used to evaluate the causal relationships in a physical system (Pearl, 2009; Oizumi et al., 2014) (see also Appendix S1). The animat is set into an initial state, a state transition of the system is observed, and then the resulting state is recorded. This process is repeated many times for all possible initial states, and the results specify the transition probability function *p* of the system. Using the transition probability functions, the values of D_1_, D_2_ and μ[Φ] were calculated exactly for each animat in the population using the methods described above.

When calculating differentiation, an important consideration is which elements should contribute to the differentiation measures. Differentiation could be made arbitrarily large by simply including additional elements in the definition of the physical system, without having any effect on the Φ values. This requires an additional exclusion assumption: only elements which are part of the complex (set of maximally irreducible elements) are included in the differentiation estimation, others have been excluded (Oizumi et al., 2014). For the current animats, all internal elements contributed to the complex of the physical system, and were used in the estimation of D_1_ and D_2_.

Elements outside the complex of a physical system are considered *background conditions* (Oizumi et al., 2014), and their state can affect both Φ and *D*. Here, the sensor and motor elements of the animats are background conditions. The values of differentiation and integrated information for each animat are calculated using only the complex (internal elements), but averaged over all possible background conditions (states of background elements).

Using the Mann-Whitney U test, μ[Φ] was significantly higher (*p* = 0.003) in the 2 sensor condition (median value of 1.434) than in the 3 sensor condition (median value of 1.077). This result was consistent with the theory developed in the Methods section, since animats with more internal elements have the potential for more integrated elements, more mechanisms, and thus greater Φ values. To control for the effect of network configuration, differentiation values were adjusted for the number of internal elements in the network using linear regression.

**Figure 3:**
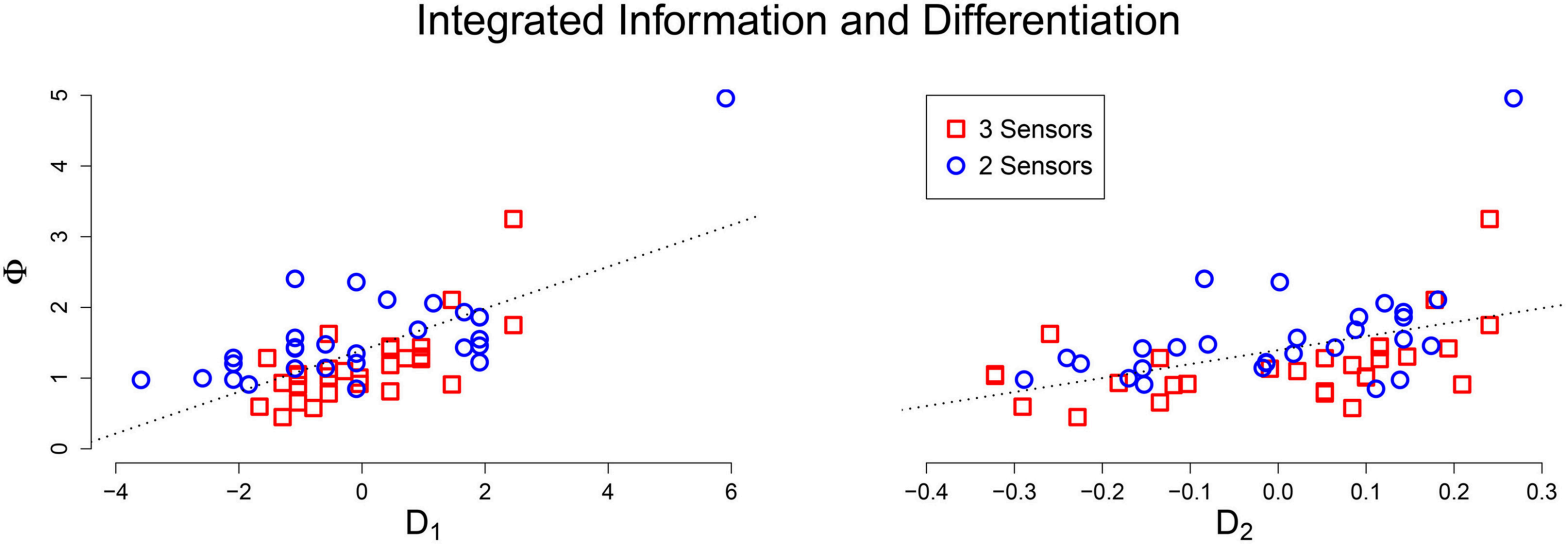
**Scatter plot of adjusted differentiation values (Left: D_1_, Right: D_2_) against average integrated information**. In both plots there is a clear positive linear relationship between *D* and Φ.

Using Pearson correlation coefficient, μ[Φ] had a significant linear relationship with D_1_ (*r* = 0.668, *p* < 0.001) and D_2_ (*r* = 0.452, *p* < 0.001). A scatter plot between integrated information and the (network size adjusted) differentiation values shows a clear linear relationship (Figure 3). This positive relationship confirms that for an integrated system, greater values of differentiation correspond to greater values of average integrated information. The remaining variability in the data is likely due to the degree of integration in the system; weakly integrated systems will fall below the line, while strongly integrated systems will be above the line. The potential outlier at the top of the plot corresponds to a system that not only has above average differentiation, but also exceptional integration, resulting in a Φ value that is far above average.

The animat systems analyzed in the current work are small in comparison to the size of system involved in neuroimaging studies; however, it is reasonable that the results may hold in larger systems. The linear relationship is equivalent for both the 3 and 4 node networks after controlling for network size. Furthermore, the networks were evolved using a genetic algorithm that involves random mutations at each stage, and thus constitute a random, independent sample. There is no reason to believe that the sample is biased toward any specific structure that would prevent the results from generalizing. One difference that may occur at larger scales is greater variability in the degree of integration of the physical system. Thus, at larger scales, we still expect the linear relationship between differentiation and integrated information to hold, but perhaps with a greater degree of variability. Of course, the extent to which the present conclusions will actually hold for larger networks will require further validation.

### Stimulus set differentiation

To measure evoked differentiation, the system of interest is observed while being presented with a sequence of stimuli. Let {**w**_*t*_} be the sequence of states the system enters during exposure to a stimulus set **W** of length *T*, and Ω_*W*_ be the set of unique states in {**w**_*t*_}. The evoked differentiation, $D_{1}^{W}$ is calculated by counting the number of unique states the system entered during exposure to the stimulus set,

$$D_{1}^{W}=|\Omega_{W}|.$$

For $D_{2}^{W}$, we first calculate the proportion of time each element was ON,

$$u_{i}^{W}=\frac{1}{T}\sum_{t=1}^{T}w_{t,i},$$

and then combined those to estimate the cumulative variance,

$$D_{2}^{W}=\sum_{i=1}^{n}u_{i}^{W}\left(1-u_{i}^{W}\right)$$

Evoked differentiation was directly influenced by the choice of stimulus set. Two different stimulus sets were presented to each animat, which can be generally described as “blocks” and “points.” The “blocks” stimulus set consisted of blocks of length 3 or 5, passing over the animats visual field from the left or right. The “points” stimulus set consisted of exactly one sensor randomly being activated at every time. Both stimulus sets consisted of *T* = 32 time points. In 34 of the animats, the number of unique states evoked was greater for the “blocks” than the “points,” in 15 the “points” evoked more unique states, and in 3 they were equal. Overall, the differentiation was greater for the “blocks” compared to the “points,” for both $D_{1}^{W_{\max}}$ (mean difference = 0.270, *p* = 0.010) and $D_{2}^{W_{\max}}$ (mean difference = 0.280, *p* < 0.001).

**Figure 4:**
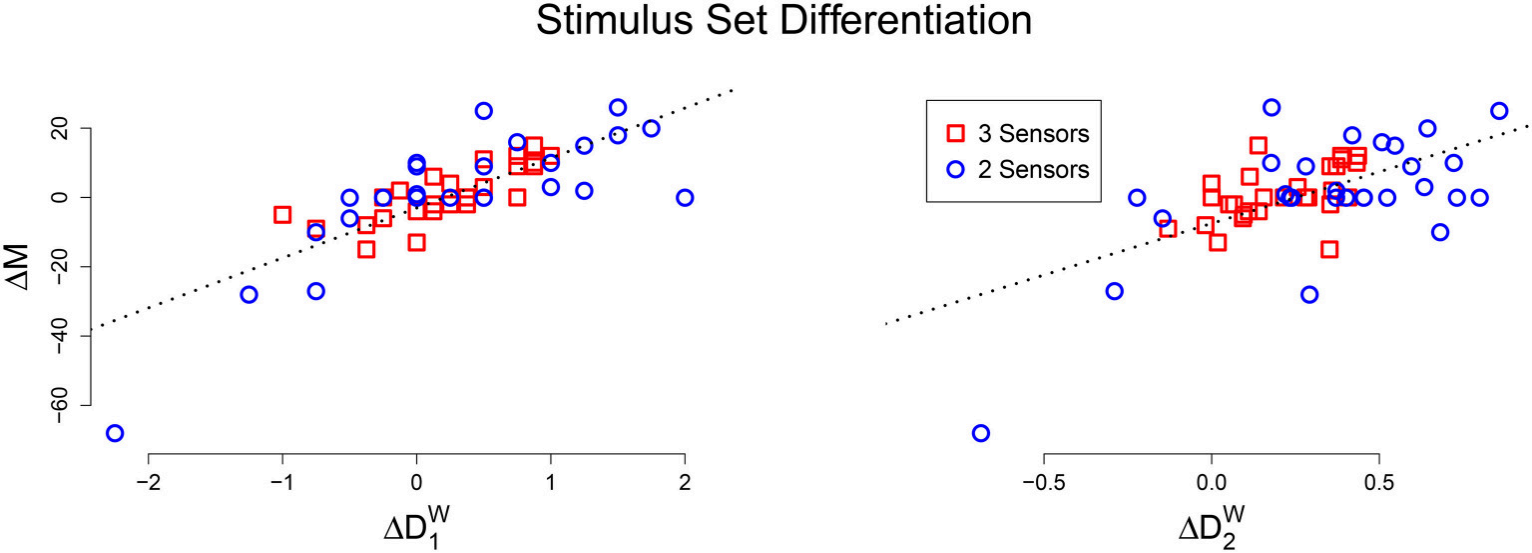
**Scatter plot of the difference in activated mechanisms and evoked differentiation between the “blocks” and “points” stimulus sets (Left: D_1_, Right: D_2_)**. In both plots there is a clear positive linear relationship.

For each animat, we also calculated the number of unique mechanisms activated by the stimulus sets, to see if there was a relationship to integrated information. A significant linear relationship is found between the difference in activated mechanisms and the difference in evoked differentiation, for both D_1_ (*r* = 0.783, *p* < 0.001) and D_2_ (*r* = 0.605, *p* < 0.001), Figure 4 is a scatter plot of the results. This means that for a particular animat, if the “blocks” stimulus set evokes greater differentiation than the “points” stimulus set, than it will also activate more mechanisms and vice versa.

The above result suggests that by presenting the animat with a sufficiently rich stimulus set, it should activate many or all of its mechanisms, which should be associated with a high value of integrated information. Longer stimulus sets of length *T* = 4608 (the number of samples used in the evolution of animats) were used in an attempt to evoke the highest levels of differentiation. Ideally, all possible stimulus sets would be considered to find the set which evokes the highest differentiation. For practical reasons, here we restricted the search to randomly generated stimulus sets with three different levels of stimulus entropy, and selected the one that forced it to enter the most states and thus activate the most mechanisms,

$$W_{\max}=\underset{W}{\arg\max}|\Omega_{W}|.$$

**Figure 5:**
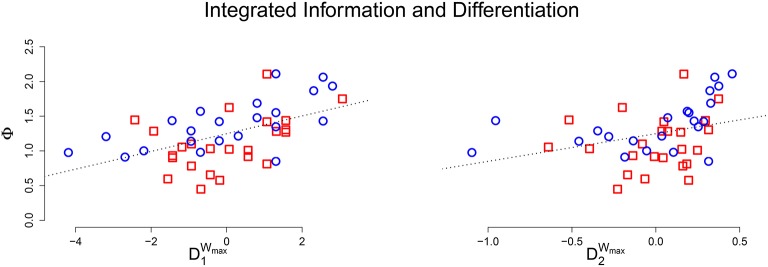
**(Top) Scatter plot of the average integrated information against estimated differentiation (Left: $D_{1}^{W_{\max}}$, Right: $D_{2}^{W_{\max}}$) for the connected group of animats**. A positive linear relationship exists for both measures, but is not as strong as in the perturbational analysis.

The maximum entropy stimulus set evoked the most unique states, and also most accurately approximate the true differentiation of the system; the average relative error was 7.8% between D_1_ and $D_{1}^{W_{\max}}$ and 12.5% between D_2_ and $D_{2}^{W_{\max}}$. The ability to approximate differentiation with stimulus set differentiation is further explored in S3, investigating the role of sample size and measurement errors. The relationship between Φ and stimulus set differentiation also mimicked the results from the perturbational analysis (*r* = 0.519, *p* < 0.001 for $D_{1}^{W_{\max}}$ and *r* = 0.334, *p* = 0.023 for $D_{2}^{W_{\max}}$). Figure 5 shows a scatter plot of μ[Φ] against stimulus set differentiation, and the linear relationship remains for both measures.

### Differentiation measures

The indices of differentiation investigated here were part of a large class of possible measures of system differentiation; they were chosen for this work because they were well suited to proving theorems that related them to integrated information and in principle lend themselves to practical and computationally efficient ways of studying consciousness. Both D_1_ and D_2_ are clearly related to other measures of differentiation that capture either the number or differences between potential states of a physical system. Several alternative measures were investigated by presenting a rich stimulus set to the animats and looking at the relationship between evoked differentiation and μ[Φ]. Measures which, like D_1_, considered the potential internal states of a system, such as state entropy (*r* = 0.475, *p* = 0.001), or Lempel-Ziv complexity (Lempel and Ziv, 1976) (*r* = 0.406, *p* = 0.006) were significantly correlated with μ[Φ]. Other measures that, like D_2_, are affected by the average distance between states, can also capture state differentiation. The average Euclidean distance had a significant relationship to μ[Φ] (*r* = 0.384, *p* = 0.011) similar to the measures used in this work. On the other hand, the correlation distance does not correlate with integrated information (*r* = 0.157, *p* = 0.315), presumably because this metric is not well suited for binary data. Measures of external system states, such as sensor entropy (*r* = −0.202, *p* = 0.193) or sensor-motor mutual information (*r* = −0.269, *p* = 0.081) showed no significant relationship to μ[Φ]. This emphasizes that Φ is a measure of intrinsic cause-effect power and is primarily concerned with internal elements, rather than stimulus or behavioral differentiation. It is worth noting that the animats in the current study had not evolved to interact with their world, and for agents that interact with their environment, such as humans, behavioral differentiation becomes more relevant and may be related to Φ (Albantakis et al., 2014).

## Discussion

We have demonstrated a theoretical link between integrated information (Φ) and measures of differentiation (*D*) of a physical system. A physical system with high values of integrated information must be constituted of many elements, have a large state space (Theorem 2.3), and have many mechanisms, including mechanisms composed of multiple elements (high-order mechanisms). Moreover, in order to have high Φ on average, it must have high differentiation (Theorem 0.4), i.e., have many potential states (D_1_), and have those states be as different as possible from each other (D_2_). Essentially, a physical system with typically large values of integrated information is necessarily one with high differentiation.

We then performed a simulation study using artificially evolved networks—animats—to more precisely determine the relationship between integrated information and differentiation. The results demonstrate that, as long as the system under consideration is integrated, then high values of D_1_ and D_2_ correspond to large values of μ[Φ] (see Figure 3). For systems connected to an external environment, we also investigated the differentiation evoked by a stimulus set. We found a positive correlation between the number of distinct mechanisms activated and the state differentiation evoked by a stimulus set (see Figure 4). Furthermore, if the stimulus set is sufficiently broad, stimulus-evoked differentiation can approximate the state differentiation of the system obtained by systematic perturbation of its internal elements (see Figure 5).

### Studying cause-effect power with state differentiation

The results of this work provide a theoretical foundation for using state differentiation to study the intrinsic causal properties of a physical system. Several important aspects were identified that should be considered when studying differentiation: integration assumption; connectedness assumption; choice of stimulus set; choice of differentiation measure. We will briefly discuss each point in the context of neuroimaging and suggest some procedures for future experimental work.

#### Integration

A system with low differentiation is also one with low integrated information, but it is not necessarily true that a system with high differentiation has high integrated information (Figure 2). For high D to predict high Φ, a system has to meet the additional requirement of being highly integrated, that is, not being easily decomposable into independent components (Tononi, 2004; Oizumi et al., 2014). A graph of the pairwise causal relationships between system elements can be used to test the integration assumption. If the graph is strongly connected (there exists a directed path between any two elements), then the system is likely integrated. Otherwise, if the integration assumption cannot be justified in this way, then alternative measures must be used which capture the integration of the system, for example Φ_*E*_ (Barrett and Seth, 2011) or Φ^*^ (Oizumi et al., 2015).

In the case of the human brain, the assumption of integration is justified by its remarkable degree of anatomical and function connectivity, especially within the cerebral cortex (Sporns et al., 2005). Thus, if the cerebral cortex of a healthy adult human shows a high value of differentiation, one can reasonably assume that it will also have a high value of integrated information which, according to integrated information theory (IIT) (Tononi, 2004; Oizumi et al., 2014), should reflect the presence of subjective experience. Supporting this notion, several empirical studies have shown a positive relation between state differentiation and consciousness across different modalities and with different measures of differentiation (Gosseries et al., 2011; Sarà et al., 2011; Barttfeld et al., 2015; Hudetz et al., 2015). It should be kept in mind, however, that IIT ascribes consciousness to the particular set of elements for which Φ reaches a maximum, which requires additional analyses and assumptions to be explored in future work.

#### Connectedness

The differentiation evoked by a stimulus set can be used to make inferences about the integrated information of a system. However, this is only possible if the system is not only integrated, but also connected to the external environment, that is, the stimuli must have a causal effect on the state of the system. The connectedness requirement ensures that the stimuli can force the system to explore its state space, and that the mechanisms it activates are “about”İ the stimuli. The connectedness assumption can be tested by measuring the system's response to different stimuli that are presumably meaningful to the system (see below): if the system's response is the same regardless of stimuli, or it is different but unrelated to the stimuli, then it is not connected. For example, phenomenally, dream experiences are highly integrated and differentiated. Experiments that directly perturb the cerebral cortex using transcranial magnetic stimulation and record its responses with high-density electroencephalography suggest that the dreaming brain supports neurophysiological activity patterns that are also integrated and differentiated (Massimini et al., 2010; see also Sarasso et al., 2015 for ketamine dreams). However, the sleeping brain is disconnected from its environment through mechanisms that block the propagation of sensory stimuli (Funk et al., 2016). As shown in Figure 2, presenting a diverse set of stimuli to a disconnected system offers no guarantee that it will explore its state space, hence differentiation may be highly underestimated. On the other hand, during wakefulness, the alert brain is both integrated and tightly connected to its environment, with which it interacts using multiple sensory modalities (vision, audition, etc.). In fact, recent neuroimaging studies have been able to exploit the differentiation evoked by a visual stimulus set to determine whether or not the stimuli were perceived (Montijn et al., 2016) as well as to compare the meaningfulness of different sets of stimuli for the subject (Boly et al., 2015).

#### Choice of stimulus set

We have demonstrated that if a system is integrated and connected to its environment (such as the awake brain), then the integrated information of the system can be estimated by exposing the system to a sufficiently rich stimulus set and measuring the evoked differentiation. The choice of stimulus set is important because it will directly impact the evoked differentiation and hence estimates of Φ. The general prescription to obtain the best approximation to state differentiation, and hence the strongest relationship to Φ, is to employ a stimulus set that is most “meaningful” to the system, in the sense that it should activate as many as possible of the system's mechanisms (Boly et al., 2015).

The animats in our study had two or three binary sensors through which they were connected to their environment. Under our controlled conditions, it was possible to present the animats with all possible stimuli (individual sensor states), as well as short sequences of stimuli (up to the limit of their internal memory). The real world is of course much richer than the animat world, ruling out the option of presenting all possible stimuli and forcing the choice of a small subset. This subset should not merely have high entropy, but should contain different stimuli that are likely to trigger different activity patterns in the system. For evolved systems, such as brains and animats, a natural choice is that of stimuli that are as varied as possible but that are sampled from the “typical” world to which the system has adapted in the course of evolution, development and learning. Such stimuli are the most meaningful to a given system and have the highest likelihood of evoking differential patterns of activity. Accordingly, recent neuroimaging studies in humans have shown that movie clips or highly meaningful slides from the natural world can evoke high neurophysiological differentiation, whereas white noise stimuli evoke minimal differentiation, despite having equal or greater stimulus entropy (Boly et al., 2015). In general, the meaningfulness of stimuli will vary to some extent from person to person, so ideally the stimulus set should be optimized based on the concepts available to each participant. In the case of animats, a fair representation of “meaningful” stimuli would include both “blocks” and “points” that the animats have evolved to catch and avoid (Albantakis et al., 2014).

Another consideration is the amount of noise, or measurement error in the data. A better estimate of differentiation can be achieved by repeating stimuli and averaging to find the deterministic component (see Appendix S3). However, there are a limited number of stimuli that can be presented in a single experiment. This creates a trade-off, between showing a more varied (and perhaps more meaningful) stimulus set and repeating stimuli to get a more accurate estimate.

#### Choice of differentiation measure

The theoretical results show that systems with high average integrated information will have high values of both measures of differentiation investigated here (D_1_, the number of potential system states; and D_2_, the variability of individual elements of the system). The simulations also indicated that D_1_ and D_2_ are strongly correlated (*r* = 0.91, *p* < 0.001), suggesting that it should be sufficient to measure only one of the two quantities. Indeed, a bootstrap hypothesis test found no significant difference in the relationship between the two differentiation quantities and integrated information (*p* = 0.170). For empirical work, the statistical properties of D_2_ (see Appendix S3) make it preferable for studying integrated information. As shown here, compared to D_1_, D_2_ is more robust to noise and requires fewer samples to get an accurate result for large networks, both factors that are particularly relevant for neuroimaging studies.

For neuronal systems, the choice of differentiation measure will depend on the mode of neuroimaging technique employed. The brain can be studied at many different spatial and temporal scales (individual spikes, calcium imaging, EEG/fMRI, etc). It is not clear what the correct scale is to study consciousness; however, IIT proposes that it should be the scale that maximizes cause-effect power (Tononi and Koch, 2015). Unfortunately, the scale that maximizes cause-effect power is not currently known, but perhaps applying the current differentiation techniques across a range of possible scales will provide some clarity to this question.

Given a specific spatio-temporal scale, the measure of state differentiation used should capture the number and/or differences between potential system states; it should not measure merely stimulus or behavioral differentiation (e.g., sensor entropy). The two measures studied in this work were selected based on their mathematical properties, so that we could analytically demonstrate the relationship between integrated information and state differentiation. However, there are other alternative options for capturing differentiation, several of which have been applied to neuroimaging data (Gosseries et al., 2011; Sarà et al., 2011; Barttfeld et al., 2015; Boly et al., 2015; Hudetz et al., 2015; Montijn et al., 2016). These alternative measures are also expected to relate to integrated information (see above), as long as the assumptions of an integrated and connected physical system are satisfied.

## Author contributions

WM and GT designed the experiment, WM derived theoretical relationships, WM and JG performed the experiment and analyzed the data, WM drafted the manuscript, all authors edited the manuscript.

### Conflict of interest statement

The authors declare that the research was conducted in the absence of any commercial or financial relationships that could be construed as a potential conflict of interest.

## Figures and Tables

**Figure A1:**
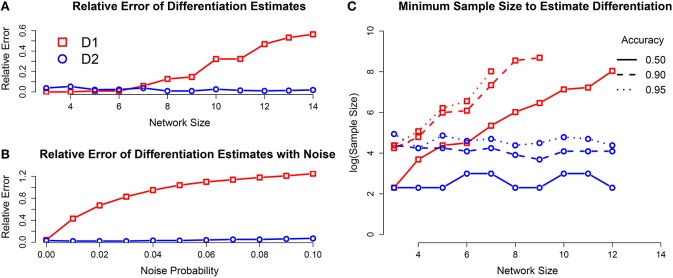
**(A)** Relative error of differentiation estimates using *T* = 4608 samples, as a function of network size. The estimates of D_1_ get worse as network size increases, but the estimates for D_2_ stay accurate. **(B)** Relative error for networks with seven internal elements with increasing levels of measurement error. The estimate of D_1_ is greatly affected by even small amounts of noise, while D_2_ has only margin increase in error. **(C)** Minimum sample size to estimate differentiation for desired accuracy. The necessary sample size for D_2_ does not increase as the network size increases, however the sample size for D_1_ increases exponentially.

## Appendix S1 - mathematical definitions

Mathematical definitions are provided for the key terms in IIT, for a complete set of definitions from the theory see Oizumi et al. (2014) and Tononi (2015).

### Earth movers distance

In IIT the Earth Movers Distance (Rubner et al., 2000) (EMD) is the metric used to compare both cause-effect repertoires and cause-effect structures. The EMD is the minimum cost of transforming one “pile of dirt” into a different “pile of dirt,” where the cost is equal to the amount of “dirt” moved multiplied by the distance it has moved.

For cause-effect repertoires the “dirt” is the probability mass at each past or future system state. The distance the “dirt” is moved, the distance between system states, is the Hamming distance. The Hamming distance between two states is equal the number of individual elements whose states differ between the two states. The Hamming distance between two binary states **s, s**^*^ ∈ Ω_*S*_ is

$$d_{ss^{*}}=\sum_{i=1}^{n}{\left(s_{i}-s_{i}^{*}\right)}^{2}.$$

For cause-effect structures, we are transforming one set of mechanisms into another set of mechanisms. The “dirt” is the integrated information (φ) of the mechanism, and the distance the “dirt” is moved is the combined EMD distance between the cause and effect repertoires of the mechanism. The distance between two mechanisms **m**_*t*_ and ${\text{m}}_{t}^{*}$ is

$$\begin{array}{l} d(m_{t},m_{t}^{*})=\text{emd}(p_{\text{cause}}(z|m_{t}),p_{\text{cause}}(z|m_{t}^{*}))\\ \;+\text{emd}(p_{\text{effect}}(z|m_{t}),p_{\text{effect}}(z|m_{t}^{*})) \end{array}$$

#### Cause-effect repertoire

Consider a candidate mechanism **M**∈ℙ(**S**) and past/future purviews **Z**_*t*±1_ ∈ ℙ(**S**).

The cause repertoire of an element of the candidate mechanism *M*_*i*_ = *m*_*i, t*_ over the past purview **Z**_*t*−1_ is the probability function for the state of the past purview conditioned on the current state of the element, evaluated causally by perturbing the system into all possible states (using the *do* operator, as defined by Pearl, 2009),

$$p_{\text{cause}}(z|m_{i,t})\equiv \frac{\sum_{z^{c}\in \Omega_{Z^{c}}}p(m_{i,t}|do(z,z^{c}))}{\sum_{s\in \Omega_{S}}p(m_{i,t}|do(s))},\;z\in \Omega_{Z_{t-1}}.$$

The inputs of every element are perturbed independently using *virtual elements* to account for the effects of common input. The resulting cause repertoire for the entire candidate mechanism has the form

$$p_{\text{cause}}(z|m_{t})\equiv \frac{1}{K}\prod_{i=1}^{\left|m_{t}\right|}p_{\text{cause}}(z|m_{i,t}),\;z\in \Omega_{Z_{t-1}},$$

where *K* is the normalization term,

$$K=\sum_{z\in \Omega_{Z_{t-1}}}\prod_{i=1}^{\left|m_{t}\right|}p_{\text{cause}}(z|m_{i,t}).$$

Similarly, the effect repertoire of the candidate mechanism in a state **M**_*t*_ = **m**_*t*_ over an element of the future purview *Z*_*i*_ ∈ **Z**_*t*+1_ is given by

$$p_{\text{effect}}(z_{i}|m_{t})\equiv \frac{1}{\left|\Omega_{M^{c}}\right|}\sum_{m^{c}\in \Omega_{M^{c}}}p(z_{i}|do(m_{t},m^{c})),\;z_{i}\in \Omega_{Z_{i}}$$

The effect repertoire for the candidate mechanism in a state **M** = **m**_*t*_ over the entire future purview **Z**_*t*+1_ is then,

$$p_{\text{effect}}(z|m_{t})\equiv \prod_{i=1}^{\left|z\right|}p_{\text{effect}}(z_{i}|m_{t}).$$

#### Mechanism cut

For a physical system **S**, candidate mechanism **M** and a past or future purview **Z**, a *cut* of (**M**, **Z**) is a partition into four sets,

$$cut=\{M^{\left(1\right)},M^{\left(2\right)},Z^{\left(1\right)},Z^{\left(2\right)}\},$$

such that **M**^(1)^ and **M**^(2)^ partition **M**, **Z**^(1)^ and **Z**^(2)^ partition of **Z**, (**M**^(1)^ ∪ **Z**^(1)^) ≠ ∅ and (**M**^(2)^ ∪ **Z**^(2)^) ≠ ∅.

The cause or effect repertoire of the *cut* candidate mechanism is found by assuming **Z**^(1)^|**M**^(1)^ and **Z**^(2)^|**M**^(2)^ are independent (the connections between them have been “cut”),

$$p_{\text{cause}}^{\text{cut}}(z|m_{t})=p_{\text{cause}}(z^{\left(1\right)}|m_{t}^{\left(1\right)})\times p_{\text{cause}}(z^{\left(2\right)}|m_{t}^{\left(2\right)}).$$

The effect-information is similarly defined as the product of the partitioned repertoires,

$$p_{\text{effect}}^{\text{cut}}(z|m_{t})=p_{\text{effect}}(z^{\left(1\right)}|m_{t}^{\left(1\right)})\times p_{\text{effect}}(z^{\left(2\right)}|m_{t}^{\left(2\right)}).$$

#### System cut

For a physical system **S**, a system cut is a directed partition,

$$cut=\{S^{\left(1\right)},S^{\left(2\right)}\},$$

such that

$$S^{\left(1\right)}\neq \emptyset ,\;S^{\left(2\right)}\neq \emptyset ,\;(S^{\left(1\right)}\cap S^{\left(2\right)})=\emptyset ,\;(S^{\left(1\right)}\cup S^{\left(2\right)})=S.$$

The cause-effect structure of the cut system is calculated from a cut transition probability function, with **S**^(1)^ → **S**^(2)^ (the connections from **S**^(1)^ to **S**^(2)^ have been injected with noise),

$$p^{\text{cut}}(s_{t}|s_{t-1})=p\left(s_{t}^{\left(1\right)}|s_{t-1}\right)\times p\left(s_{t}^{\left(2\right)}|s_{t-1}^{\left(2\right)}\right)$$

### Appendix S2 - proof of theorems

The EMD between repertoires is defined as least amount of work necessary to transform one repertoire into the other. One possible transformation is to distribute the probabilities from the first repertoire to each state in the second repertoire, proportional to the probabilities in the second repertoire. This is unlikely to be the optimal transformation, but it does provide an upper bound on the EMD,

$$\text{emd}(p_{1},p_{2})\leq \sum_{s_{1}}\sum_{s_{2}}p(s_{1})p(s_{2})d_{s_{1}s_{2}},$$

where *d*_*s*_1___*s*_2__ is the distance between states *s*_1_ and *s*_2_. We shall use the symbol (^**^) to note when we apply this result.

**Theorem 2.1.** *For a physical system in a state* **S** = **s**_*t*_ ∈ Ω_*S*_ *with n binary elements, the cause and effect information of a mechanism in a state* **M** = **m**_*t*_ *are bounded*,

$$\varphi_{\text{cause}}(m_{t})\;\leq \;\frac{n}{2},\text{ and }\varphi_{\mathrm{effect}}(m_{t})\;<\;n.$$

*Proof.* For the cause information of **m**_*t*_,

$$\begin{array}{l} ci(m_{t})=\text{emd}\left(p_{\text{cause}}(z|\emptyset ),p_{\text{cause}}(z|m_{t})\right)\\ \;(**)\leq \sum_{s^{*}\in \Omega_{S}}\sum_{s\in \Omega_{S}}p_{\text{cause}}(s^{*}|\emptyset )p_{\text{cause}}(s|s_{t})d_{ss^{*}}\\ \;=\frac{1}{2^{n}}\sum_{s\in \Omega_{S}}p_{\text{cause}}(s|s_{t})\sum_{s^{*}\in \Omega_{S}}d_{ss^{*}}\\ \;=\frac{1}{2^{n}}\sum_{s\in \Omega_{S}}p_{\text{cause}}(s|s_{t})n2^{n-1}\\ \;=\frac{n}{2}\sum_{s\in \Omega_{S}}p_{\text{cause}}(s|s_{t})\\ \;=\frac{n}{2} \end{array}$$

For the effect information of **m**_*t*_,

$$\begin{array}{l} ei(m_{t})=\text{emd}\left(p_{\text{effect}}(s|m_{t}),p_{\text{effect}}(s|\emptyset )\right)\\ \;(**)\leq \sum_{s^{*}\in \Omega_{S}}\sum_{s\in \Omega_{S}}p_{\text{effect}}(s^{*}|\emptyset )p_{\text{effect}}(s|s_{t})d_{ss^{*}}\\ \;\leq n\sum_{s^{*}\in \Omega_{X}}p_{\text{effect}}(s^{*}|\emptyset )\sum_{s\in \Omega_{S}}p_{\text{effect}}(s|s_{t})\\ \;=n \end{array}$$

                        □

**Corollary 2.2.** *For a physical system in a state* **S** = **s**_*t*_ ∈ Ω_*S*_ *with *n* binary elements, and mechanism **M** = **m**_*t*_, the integrated information of **m**_*t*_ is bounded by*

$$\varphi^{\max}(m_{t})\leq \frac{n}{2}.$$

*Proof*. For a fixed past purview **Z**_*t*−1_, note that by cutting all connections between mechanism and purview, the cut repertoire is simply the unconstrained repertoire, so we can apply Theorem 2.1.

$$\begin{array}{l} \text{ emd}(p_{\text{cause}}(z|m_{t}),p_{\text{cause}}^{\text{MIP}}(z|m_{t}))\\ \leq \text{emd}(p_{\text{cause}}(z|m_{t}),p_{\text{cause}}(z|\emptyset ))\\ =ci(m_{t})\\ \leq \frac{\left|Z_{t-1}\right|}{2} \end{array}$$

Since the integrated information is the maximum across all possible purviews,

$$\begin{array}{l} \varphi^{\max}(m_{t})\leq \varphi_{\text{cause}}(m_{t})\\ \;\leq \underset{Z_{t-1}}{\max}\frac{\left|Z_{t-1}\right|}{2}\\ \;\leq \frac{n}{2} \end{array}$$

                        □

**Theorem 2.3.** *For a physical system in a state* **S** = **s**_*t*_ ∈ Ω_*S*_ *with *n* binary elements, the integrated information of its cause-effect structure is bounded by*

$$\Phi (s_{t})\leq \frac{3n^{2}(2^{n}-1)}{4}.$$

Proof.

$$\begin{array}{l} \;\Phi (s_{t})=\text{emd}(C(s_{t}),C(s_{t}^{\text{MIP}}))\\ \;\leq \sum_{m\in C(s_{t})}\varphi (m)(ci(m)+ei(m))\\ \left(Thm2.1)=\sum_{m\in C(s_{t})}\frac{n}{2}(ci(m)+ei(m)\right)\\ (Cor2.2)\leq \sum_{m\in C(s_{t})}\frac{n}{2}\left(\frac{n}{2}+n\right)\\ \;\leq |C(s_{t})|\frac{3n^{2}}{4}\\ \;\leq (2^{n}-1)\frac{3n^{2}}{4}. \end{array}$$

                        □

**Theorem 2.4.** *For a physical system* **S** *with* n *binary elements, state space* Ω(**S**) *and candidate mechanism* **M** ⊆ **S**, *the average cause and effect information are bounded,*

$$\mu [ci(m)]\;\leq \;\frac{nD_{1}}{2^{n+1}}\;\leq \;\frac{n}{2},\text{ and }\mu [ei(m)]\;\leq \;2D_{2}\;\leq \;\frac{n}{2}.$$

*Proof.* The result of cause information follows directly from Theorem 2.1, since cause information has the same bounded for each state, the average must be similarly bounded.

$$\begin{array}{l} \mu [ci(m)]=\sum_{s\in \Omega }\frac{1}{\left|\Omega \right|}ci\left(s\right)\\ \;=\sum_{s\in \hat{\Omega }}\frac{1}{\left|\Omega \right|}ci\left(s\right)\\ \;\leq \sum_{s\in \hat{\Omega }}\frac{1}{\left|\Omega \right|}\frac{n}{2}\\ \;=\frac{\left|\hat{\Omega }\right|}{\left|\Omega \right|}\frac{n}{2}\\ \;=\frac{nD_{1}}{2^{n+1}}\\ \;\leq \frac{n}{2} \end{array}$$

For the result on effect information, we define some preliminary notation. Enumerate the states of the system {**s**_j_, *j* = 1…2^*n*^} = Ω*S*, then define *q*_*k*_ = *p*_*effect*_(**s**_*k*_|∅) (the unconstrained effect probability of state *s*_*k*_) and *p*_*jk*_ = *p*_effect_(**s**_*k*_|**s**_*j*_) (the effect probability of state *s*_*k*_ constrained on the current state being *s*_*j*_). Furthermore, define *J*_*i*_ = {*j*|*s*_*j, i*_ = 1}, the set of all states *s*_*j*_ such that *i*^*th*^ element of **s**_*j*_ is ON (1). The unconstrained probability that element *i* is ON (1) is then,

$$u_{i}=\sum_{j\in J_{i}}q_{j}.$$

Finally, define *v*_*j, i*_ to be the unconstrained effect probability that element *i* will be the same as it is for state **s**_*j*_,

$$v_{j,i}=\{\begin{array}{l} u_{i}\text{ if }s_{j,i}=1\\ 1-u_{i}\text{ otherwise}. \end{array}$$

$$\begin{array}{l} \mu [ei(m)]=\sum_{i=1}^{2^{n}}\frac{1}{\left|\Omega_{S}\right|}ei(s_{i})\\ \;(**)\leq \sum_{i=1}^{2^{n}}\frac{1}{2^{n}}\sum_{j=1}^{2^{n}}q_{j}\sum_{i=k}^{2^{n}}p_{ik}d_{s_{j}s_{k}}\\ \;=\sum_{j=1}^{2^{n}}q_{j}\sum_{i=1}^{2^{n}}\sum_{k=1}^{2^{n}}\frac{p_{ik}d_{s_{j}s_{k}}}{2^{n}}\\ \;=\sum_{j=1}^{2^{n}}q_{j}\left(n-\sum_{m=1}^{n}v_{j,i}\right)\\ \;=n-\sum_{i=1}^{n}\sum_{j=1}^{2^{n}}q_{j}v_{j,i}\\ \;=n-\sum_{i=1}^{n}\left(\sum_{j\in J_{i}}q_{j}u_{i}+\sum_{j\in J_{i}^{c}}q_{j}(1-u_{i})\right)\\ \;=n-\sum_{i=1}^{2^{n}}u_{i}^{2}+{\left(1-u_{i}\right)}^{2}\\ \;=2\sum_{i=1}^{n}u_{i}(1-u_{i})\\ \;=2D_{2}\\ \;\leq \frac{n}{2} \end{array}$$

                        □

**Theorem 2.5.** *For a physical system* **S** *with *n* binary elements, and corresponding state space* Ω_*S*_ = {0, 1}^*n*^, *the average integrated information is bounded by*

$$\mu [\Phi ]=\sum_{s\in \Omega_{S}}\frac{\Phi (s)}{\left|\Omega_{S}\right|}\leq (2^{n}-1)\frac{n}{2}\left(\frac{nD_{1}}{2^{n+1}}+2D_{2}\right)\leq \frac{(2^{n}-1)n^{2}}{2}.$$

Proof.

$$\begin{array}{l} \;\mu_{\Phi }=\sum_{s\in \Omega_{S}}\frac{\Phi (s)}{\left|\Omega_{S}\right|}\\ \;\leq \sum_{s\in \Omega_{S}}\frac{1}{\left|\Omega_{S}\right|}\sum_{m\subseteq S}\varphi (m)(ci(m)+ei(m))\\ \left(Cor2.2)\leq \sum_{m\subseteq S}\frac{n}{\left(2|\Omega_{S}|\right)}\sum_{s\in \Omega_{S}}(ci(m)+ei(m)\right)\\ \;\leq \sum_{m\subseteq S}\frac{n}{2}\left(\sum_{s\in \Omega_{S}}\frac{ci(m)}{\left|\Omega_{S}\right|}+\sum_{s\in \Omega_{S}}\frac{ei(m)}{\left|\Omega_{S}\right|}\right)\\ \;=\sum_{m\subseteq S}\frac{n}{2}\left(\mu [ci(m)]+\mu [ei(m)]\right)\\ (Thm2.3)\;\leq \sum_{m\subseteq S}\frac{n}{2}\left(\frac{nD_{1}}{2^{n+1}}+2D_{2}\right)\\ \;\leq (2^{n}-1)\frac{n^{2}}{2}. \end{array}$$

                        □

**Theorem 2.6.** *Consider the integrated information for a random state of a physical system. If* Φ ∝ ∑ φ *and* σ[Φ] = *o*(μ[Φ]), *then for any* ϵ > 0 *and* δ > 0 *there exists* μ_0_ *such that for all systems with* μ[Φ] > μ_0_,

P(|Φ−μ[Φ]|≥δμ[Φ])≤ϵ.

*Proof.* Define *X* as the sum of integrated information of each candidate mechanism φ_*i*_ (*i* = 1…2^*n*^ − 1) for a random state of a physical system,

$$X=\sum_{i=1}^{2^{n}}\varphi_{i}.$$

Since Φ ∝ ∑φ, there exists *c* such that μ[Φ] = *cE*(*X*) and σ^2^[Φ] = *c*^2^*Var*(*X*).

By Chebyshev's inequality,

$$P\left(\left|X-\mu [X]\right|\geq k\sigma [X]\right)\leq \frac{1}{k^{2}},$$

so that

$$P\left(\left|c\Phi -c\mu [\Phi ]\right|\geq kc\sigma [\Phi ]\right)\leq \frac{1}{k^{2}}.$$

Taking $k=\frac{\delta \mu [\Phi ]}{\sigma [\Phi ])}$,

$$P\left(\left|\Phi -\mu (\Phi )\right|\geq \delta \mu [\Phi ]\right)\leq \frac{1}{\delta^{2}}{\left(\frac{\sigma [\Phi ]}{\mu [\Phi ]}\right)}^{2},$$

and since σ[Φ] = o(μ[Φ]), there exists μ_0_ such that for all μ[Φ] > μ_0_,

P(|Φ−μ[Φ]|≥δμ[Φ])≤ϵ.

                        □

The first assumption is that Φ is proportional to ∑φ. The population of high Φ animats used in this work support this assumption: the correlation between μ[Φ] and μ[∑φ] was ρ = 0.900 (*p* < 10^−16^). The second assumption is that μ[Φ] is greater than σ[Φ]. As *n* increases, the mean μ[Φ] grows faster than the standard deviation σ[Φ], this is also supported by the animat used in this work: there is a positive correlation between φ values, which causes the variance of ∑φ and thus also Φ to grow at a reduced rate. Thus for large systems, this seems like a reasonable assumption.

### Appendix S3 - approximating differentiation by stimulus manipulation

To explore the possibility of estimating differentiation with evoked differentiation, we evolved a population of animats that had many different states and were connected to the environment. To accomplish this, the fitness function used in the evolution was the product of the number of connections from sensor elements to internal elements, and the number of internal element states observed during exposure to a stimulus set. This resulted in animats which whose states were affected by an external world and had a large state space. There were 60 animats total for this simulation, each had two sensors and two motors, and the number of internal elements was evenly distributed between three and fourteen.

To calculate evoked differentiation, the system of interest is observed while being presented with a sequence of stimuli. Our goal is to estimated the true differentiation of the system, so we exposed the animats to several stimulus sets selected the one that forced it to best explore its state space (enter the most unique states). A maximum entropy stimulus set resulted in the greatest number of states visited and was used to estimate differentiation. A stimulus set of length *T* = 4608 (the number of samples used in the evolution of animats) was used to estimate differentiation for each of the animats, and the relative error was calculated for both measures of differentiation,

$$E_{i}=\frac{\left|D_{i}-{\hat{D}}_{i}\right|}{D_{i}},\;i=1,2.$$

For network sizes of up to seven internal elements, both differentiation quantities were estimated within an error margin of 5%. For networks with greater than seven internal elements, D_2_ is still accurately estimated, but the estimates for D_1_ get worse as the network size increases (see Figure A1A). The number of samples required to get an accurate estimate of D_1_ increased exponentially with network size, while it remained constant for D_2_ (see Figure A1C).

It is also of interest whether the differentiation can be estimated in the presence of measurement errors. We investigated this question by including measurement error into the state observations during external perturbation. For every observed state of the system, each element independently had a fixed probability of being put into the opposite binary state. The result was that the accuracy of the D_2_ estimator only decreased slightly with the inclusion of noise, while the accuracy for D_1_ decreased drastically for even small amounts of noise (see Figure A1B).

In summary, assuming the network is connected to an external world, both measures of differentiation can be accurately approximate by differentiation to a sufficiently large and rich stimulus set. The sample size necessary for accurate estimation of D_1_ increases exponentially with network size, and the accuracy is greatly reduced by measurement errors in the state observations. The estimator for D_2_ is a more robust quantity, giving quality results for large networks and in the presence of measurement errors. Thus D_2_ is preferred over D_1_ for neuronal and similar applications.
